# Novel Photosensitizer β-Mannose-Conjugated Chlorin e6 as a Potent Anticancer Agent for Human Glioblastoma U251 Cells

**DOI:** 10.3390/ph13100316

**Published:** 2020-10-16

**Authors:** Yo Shinoda, Kohei Kujirai, Kohei Aoki, Mai Morita, Masato Masuda, Lihao Zhang, Zhou Kaixin, Akihiro Nomoto, Tsutomu Takahashi, Yayoi Tsuneoka, Jiro Akimoto, Hiromi Kataoka, Rioko Rachi, Atsushi Narumi, Tomokazu Yoshimura, Shigenobu Yano, Yasuyuki Fujiwara

**Affiliations:** 1 Department of Environmental Health, School of Pharmacy, Tokyo University of Pharmacy and Life Sciences, 1432-1 Horinouchi, Hachioji, Tokyo 192-0392, Japan; y151061@toyaku.ac.jp (K.K.); kohe1170928@gmail.com (K.A.); ma131.news2@gmail.com (M.M.); tsutomu@toyaku.ac.jp (T.T.); tsuneoka@toyaku.ac.jp (Y.T.); 2 Department of Applied Chemistry, Graduate School of Engineering, Osaka Prefecture University, 1-1 Gakuen-cho, Naka-ku, Sakai, Osaka 599-8531, Japan; szb02120@edu.osakafu-u.ac.jp (M.M.); syb02151@edu.osakafu-u.ac.jp (L.Z.); zhx245259630@gmail.com (Z.K.); nomoto@chem.osakafu-u.ac.jp (A.N.); 3 Department of Neurosurgery, Tokyo Medical University, 6-7-1 Nishi-Shinjuku, Shinjuku, Tokyo 160-0023, Japan; jiroaki@gmail.com; 4 Department of Gastroenterology and Metabolism, Nagoya City University Graduate School of Medical Sciences, 1 Kawasumi, Mizuho-cho, Mizuho-ku, Nagoya 467-8601, Japan; hkataoka@med.nagoya-cu.ac.jp; 5 Department of Organic Materials Science, Graduate School of Organic Materials Science, Yamagata University, Jonan 4-3-16, Yonezawa 992-8510, Japan; tcf00071@st.yamagata-u.ac.jp (R.R.); narumi@yz.yamagata-u.ac.jp (A.N.); 6 KYOUSEI Science Center for Life and Nature, Nara Women’s University, Kitauoya-Higashimachi, Nara 630-8506, Japan; yoshimura@cc.nara-wu.ac.jp (T.Y.); yano-s@cc.nara-wu.ac.jp (S.Y.)

**Keywords:** β-mannose-conjugated chlorin e6, talaporfin sodium, glioblastoma, U251, photodynamic therapy, PDT

## Abstract

A photosensitizer is a molecular drug for photodynamic diagnosis and photodynamic therapy (PDT) against cancer. Many studies have developed photosensitizers, but improvements in their cost, efficacy, and side effects are needed for better PDT of patients. In the present study, we developed a novel photosensitizer β-mannose-conjugated chlorin e6 (β-M-Ce6) and investigated its PDT effects in human glioblastoma U251 cells. U251 cells were incubated with β-M-Ce6, followed by laser irradiation. Cell viability was determined using the Cell Counting Kit-8 assay. The PDT effects of β-M-Ce6 were compared with those of talaporfin sodium (TS) and our previously reported photosensitizer β-glucose-conjugated chlorin e6 (β-G-Ce6). Cellular uptake of each photosensitizer and subcellular distribution were analyzed by fluorescence microscopy. β-M-Ce6 showed 1000× more potent PDT effects than those of TS, and these were similar to those of β-G-Ce6. β-M-Ce6 accumulation in U251 cells was much faster than TS accumulation and distributed to several organelles such as the Golgi apparatus, mitochondria, and lysosomes. This rapid cellular uptake was inhibited by low temperature, which suggested that β-M-Ce6 uptake uses biological machinery. β-M-Ce6 showed potent PDT anti-cancer effects compared with clinically approved TS, which is a possible candidate as a next generation photosensitizer in cancer therapy.

## 1. Introduction

Photodynamic therapy (PDT) is a less invasive treatment for cancer [1,2,3,4]. PDT uses a photosensitizer that accumulates in cancer cells and a harmless laser to elicit the photosensitizer to produce reactive oxygen species (ROS) to kill cancer cells [1,5,6]. Many photosensitizers for PDT have been developed, but effective photosensitizers with low side effects and costs are needed for better therapeutic effects in patients. 

Talaporfin sodium (TS, NPe6, mono-L-aspartyl chlorin e6, Laserphyrin^®^) is a second generation photosensitizer approved by the Japanese Ministry of Health, Labor, and Welfare and clinically used in PDT for early-stage lung cancer, primary malignant brain tumors, and locally remnant recurrent esophageal cancer in Japan [7,8,9,10,11,12]. TS shows fast clearance from the body and relatively short-term side effects, such as skin photosensitivity, compared with the first generation clinically approved photosensitizer porfimer sodium (Photofrin) [13]. 

**Figure 1:**
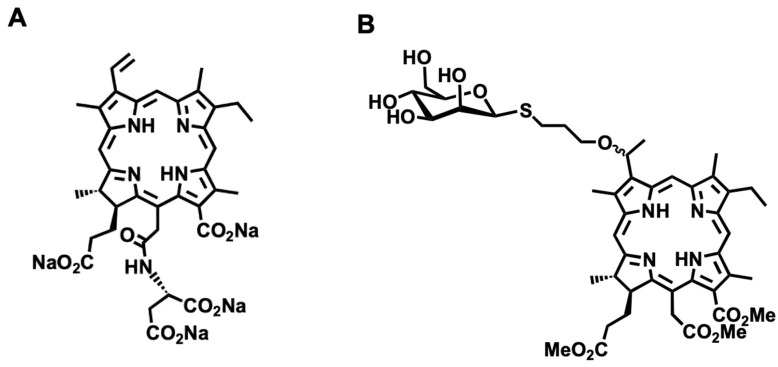
Chemical structures of talaporfin sodium (TS) and β-mannose-conjugated chlorin e6 (β-M-Ce6). (**A**) TS. (**B**) β-M-Ce6.

Recently, we developed β-glucose-conjugated chlorin e6 (β-G-Ce6) as a next generation photosensitizer [14]. It shows drastic anti-tumor effects in normal immortalized esophageal epithelial cells and esophageal cancer cells both in vitro and in vivo. Glucose conjugation is based on Warburg effects whereby cancer cells in general consume more glucose than normal cells [15,16]. Another strategy to enhance the cancer specificity of a photosensitizer is targeting tumor-associated macrophages (TAMs), a class of immune cells that exist in high numbers in the solid tumor microenvironment [17,18,19]. TAMs with high expression of mannose receptor are pro-tumorigenic cells that negatively affect therapy responsiveness [17,18,19,20]. In this context, we also developed a mannose-conjugated chlorin derivative, H2TFPC-SMan, 5,10,15,20-tetrakis (4-(α-d-mannopyranosylthio)-2,3,5,6-tetrafluorophenyl)-2,3-(methano (*N*-methyl) iminomethano) chlorin, which shows significant anti-cancer effects and accumulation in M2-polarized macrophages [21,22]. In the present study, we synthesized novel β-mannose-conjugated chlorin e6 (β-M-Ce6) as a photosensitizer derived from the core structure of chlorin e6 (Figure 1). We compared the anti-cancer effects of β-M-Ce6 with those of TS and our previously reported photosensitizer β-G-Ce6 [14] in human glioblastoma cell line U251.

## 2. Results

### 2.1. Potent Anti-Cancer Effects of β-M-Ce6 Compared with TS

**Figure 2:**
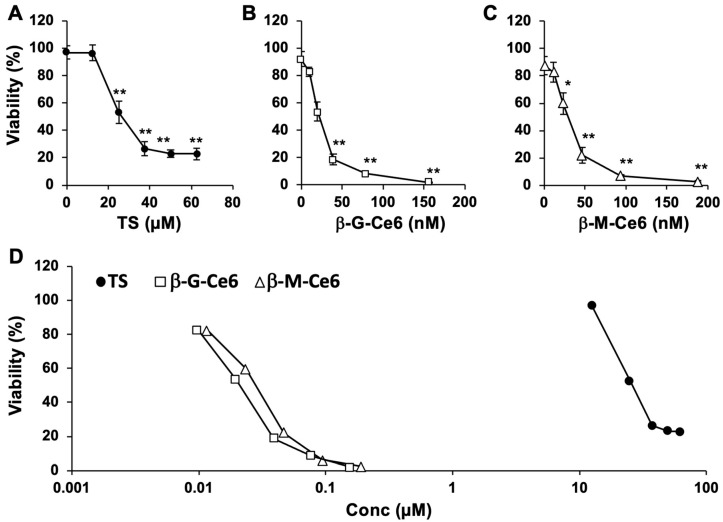
Dose dependency of anti-cancer effects for 1 h treatments. (**A**) Talaporfin sodium (TS), (**B**) β-glucose-chlorin e6 (β-G-Ce6), and (**C**) β-mannose-chlorin e6 (β-M-Ce6). (**D**) Semi-log graph of all tested compounds. *n* = 6, 6, and 8 for each compound and condition, respectively. * *p* < 0.05 and ** *p* < 0.01 vs. 0 µM of each photosensitizer, one-way analysis of variance (ANOVA) with the Tukey–Kramer post-hoc test.

We first compared the anti-cancer effects of β-M-Ce6 with those of TS and β-G-Ce6 as PDT photosensitizers on the viability of human glioblastoma U251 cells. The cells were treated with several concentrations of each photosensitizer for 1 h and then irradiated by a 664 nm laser (1 J/cm^2^) (Figure 2). Photosensitizer-treated cells without irradiation did not show any cell death (data not shown). However, laser-irradiated cells showed significant cell death after photosensitizer treatment in a dose-dependent manner. Importantly, the median lethal dose (LD_50_) of β-M-Ce6 (30 nM) was 1000× lower than that of TS (26 µM) (Figure 2D) and similar to that of β-G-Ce6 (21 nM), as reported previously [14]. These data suggest that β-M-Ce6 has potent anti-cancer effects compared with TS.

### 2.2. Fast Cellular Accumulation Property of β-M-Ce6

**Figure 3:**
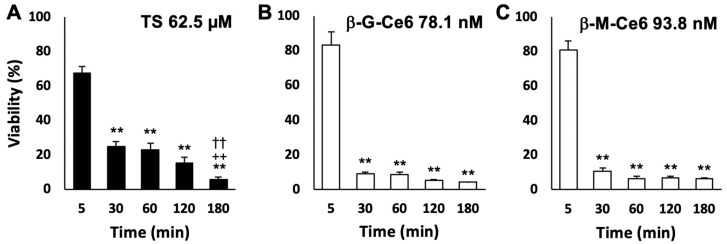
Time dependency of treatments. Doses that resulted in ~5% viability at 180 min of treatment were used. (**A**) Talaporfin sodium (TS, 62.5 µM), (**B**) β-glucose-chlorin e6 (β-G-Ce6, 78.1 nM), and (**C**) β-mannose-chlorin e6 (β-M-Ce6, 93.8 nM). *n* = 6, 6, and 8 for each compound and condition, respectively. ** *p* < 0.01 vs. 5 min, ^++^ *p* < 0.01 vs. 30 min, and ^††^ *p* < 0.01 vs. 60 min of each photosensitizer, one-way analysis of variance (ANOVA) with the Tukey–Kramer post-hoc test.

Next, we investigated the cellular accumulation rate of each photosensitizer by cell viability assays (Figure 3). The concentrations of each photosensitizer we used in this experiment (62.5 µM for TS, 78.1 nM for β-G-Ce6, and 93.8 nM for β-M-Ce6) were determined by a preliminary experiment that showed around 5% cell viability at 180 min of treatment with each photosensitizer. Using these concentrations, we performed the cellular accumulation assay of each photosensitizer revealed by changing the incubation time from 5 to 180 min. TS treatment resulted in significant cell death (24.9% viability) at 30 min of treatment and the maximal effect (5.7% viability) at 180 min of treatment. However, the maximal effects (5–10% viabilities) of β-M-Ce6 and β-G-Ce6 were already obtained at 30 min of treatment and sustained until 180 min (Figure 3). These data suggest that β-M-Ce6 has faster cellular accumulation than TS and the rate is at least six times faster than that of TS.

### 2.3. Fast Cellular Accumulation of β-M-Ce6 is Mainly Caused by Biological Machinery

**Figure 4:**
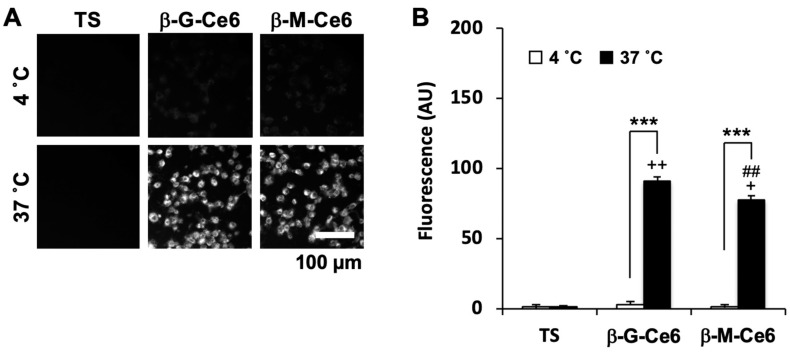
Accumulation of photosensitizers. (**A**) Representative fluorescence micrographs. (**B**) Mean fluorescent intensities of each photosensitizer (2 µM, 30 min) and temperature condition (4 and 37 °C), *n* = 40 for each photosensitizer and condition. *** *p* < 0.001, Student’s t-test. ^+^ *p* < 0.05 and ^++^ *p* < 0.01 vs. 37 °C for TS and ^##^ *p* < 0.01 vs. 37 °C for β-G-Ce6, one-way analysis of variance (ANOVA) with the Tukey–Kramer post-hoc test.

There are two major factors for cellular accumulation of a substrate. One is the physical property of the substrate, especially the partition coefficient. The other is biological machinery such as influx/efflux transporters, endo-/exo-cytosis, and several metabolic enzymes. To investigate whether the physical property of them or biological machinery were responsible for the fast cellular accumulation of β-M-Ce6, we first measured the partition coefficient (*log P*) defined by Equation (1):

(1) $$\log P=\;\log\frac{\left[C_{1-octanol}\right]}{\left[C_{PBS}\right]}$$

where [*C*_1-*octanol*_] and [*C_PBS_*] denote the concentrations of the photosensitizers being portioned into the 1-octanol phase and the phosphate buffered saline (PBS) phase, respectively. The *log P*-values of β-M-Ce6 and β-G-Ce6 were 1.88 and 1.89, respectively. In contrast, the value of TS was less than −3.00 (pH 8–12), in accordance with its pharmaceutical interview form that supplements the package insert and is provided only in Japanese. These data suggested that β-M-Ce6 had the potential to more easily accumulate and penetrate lipid bilayer than TS. Biological machinery acts properly at the appropriate temperature. Therefore, if accumulation of β-M-Ce6 used biological machinery, its accumulation would be suppressed by a low temperature. We performed a photosensitizer transport assay at normal (37 °C) and cold (4 °C) temperatures and their accumulations were analyzed by fluorescence microscopy (Figure 4). TS showed almost no fluorescence under this experimental condition (2 µM, 30 min of exposure) at both temperatures. This result was consistent with the result of cell viability assays (Figure 2). β-M-Ce6 and β-G-Ce6 showed bright fluorescence at 37 °C, but the fluorescence of each photosensitizer was significantly lower at 4 °C. Taken together, these data suggested that the fast cellular accumulations of β-M-Ce6 and β-G-Ce6 were mainly mediated by biological machinery.

### 2.4. β-M-Ce6 Accumulates in Lipid-Containing Organelles Such as the Golgi Apparatus, Mitochondria, and Lysosomes

**Figure 5:**
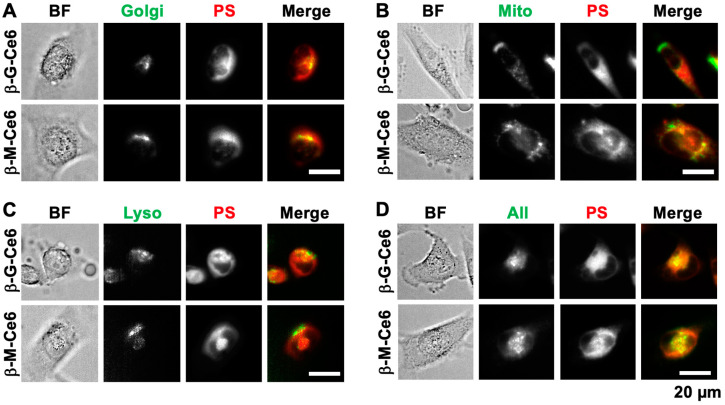
Representative images of the subcellular distribution of each photosensitizer. Brightfield (BF), organelle markers (Golgi apparatus (Golgi), Mitochondria (Mito), Lysosomes (Lyso), and co-staining of all markers (All)), 2 µM photosensitizer (PS), and merged images (green for organelle markers and red for PS) are shown. The distributions of photosensitizers compared with (**A**) the Golgi apparatus, (**B**) mitochondria, (**C**) lysosomes, and (**D**) all markers.

Subcellular distribution of a photosensitizer affects its PDT efficiency. Because of their photodynamic effect, ROS production and subsequent destruction of biological molecules, such as proteins, lipids, and nucleotides, are affected by the position of the photosensitizer. Therefore, we investigated the localization of photosensitizers by co-staining of several organelle markers and photosensitizers (Figure 5). Interestingly, all conjugated chlorins were distributed to all stained organelles such as the Golgi apparatus (Figure 5A), mitochondria (Figure 5B), and lysosomes (Figure 5C). Such phenomena were confirmed by simultaneous staining of all markers together with photosensitizers (Figure 5D). These data suggested that β-M-Ce6 and β-G-Ce6 were distributed to all lipid-containing organelles and this feature may be caused by their lipophilic properties.

## 3. Discussion

In the present study, we demonstrated that novel β-M-Ce6 has a potent anticancer effect in human glioblastoma U251 cells. This anti-cancer effect of β-M-Ce6 was 1000× higher than that of TS, a clinically approved PDT drug in Japan. The cellular uptake rate of β-M-Ce6 was at least six times faster than that of TS and its uptake may employ biological machinery. Furthermore, the subcellular distribution of β-M-Ce6 may depend on its physical properties such as a high partition coefficient.

Compared with TS, β-M-Ce6 has two large different molecular regions. One molecular region of TS has four sodium carbonate regions, but β-M-Ce6 has three methyl ester regions. This difference makes β-M-Ce6 more lipophilic than TS. Consistently, the partition coefficient of β-M-Ce6 was much higher than that of TS. This *log* *P*-value of β-M-Ce6 (1.88) was reasonable compared with our previously reported chlorin derivative that has four glucosyl residues and a *log* *P*-value of 0.13 [23]. Lipophilicity is thought to simply increase cellular permeability and enhancement of lipophilicity is usually used in prodrug design [24]. In the present study, this physical feature of β-M-Ce6 may not largely affect intracellular uptake, but affect subcellular accumulation. A low temperature significantly decreased cellular uptake of β-M-Ce6, which indicated that uptake of β-M-Ce6 may employ biological machinery. However, β-M-Ce6 showed subcellular localization in not only the plasma membrane, but also several organelles such as the Golgi apparatus, mitochondria, and lysosomes. All these cellular structures have lipid bilayers. Therefore, β-M-Ce6 localization to these subcellular regions may be explained by its physical features, lipophilicity. Furthermore, this accumulative feature of β-M-Ce6 to several organelles may enhance PDT by simultaneous degeneration of organelle functions.

The other different molecular region of β-M-Ce6 compared with TS is a mannose residue. This approach was based on the high expression of the mannose receptor in TAMs [17,19,20,25]. TAMs influence progression, metastasis, and tumor recurrence, which originate mainly from circulating monocytes, but resident macrophages also develop in a tumor [26,27]. Therefore, mannose residues may enhance accumulation of a drug in a tumor by targeting the mannose receptor expressed on TAMs [28,29]. In the present study, we did not investigate whether β-M-Ce6 accumulates in TAMs or macrophages. However, it is noteworthy that the mannose conjugation enhanced the PDT effects in glioblastoma cells. One of the effects is thought to be associated with highly expressed sugar transporters in tumors [30,31]. Fourteen glucose transporters (GLUT) and 12 sodium-glucose cotransporters (SGLT) have been reported in humans, and at least GLUT1-3 and SGLT4/5 have some affinity for mannose [30,32,33]. Therefore, the expression of these glucose transporters may explain the potent anti-cancer effects of β-M-Ce6. In addition to glucose transporters, several proteins have the potential to bind mannose and affect cellular uptake [34,35]. Indeed, U251 cells express several genes encoding proteins that potentially bind to mannose, which include 16 genes that have mannose in their gene name (GSM723932; DNA microarray study deposited in Gene Expression Omnibus (http://www.ncbi.nlm.nih.gov/geo/)). In particular, mannose receptor C type 2 and mannose-6-phosphate receptor expressed in U251 cells are possible candidates to enhance PDT effects via mannose residues. In addition to these mannose-associated proteins, other candidates may exist. Therefore, the possible mechanism of enhanced PDT effects by mannose conjugation should be clarified in a future study.

Taken together, our results strongly suggest that β-M-Ce6 has potent PDT effects compared with clinically approved TS and potential as a next generation photosensitizer. Further investigation to reveal the mechanisms and possible side effects and an in vivo study should be required for future clinical trials.

## 4. Materials and Methods 

### 4.1. Synthesis of β-M-Ce6

1-thio-β-d-tetraacetylmannose and 3-(3-bromopropoxy) chlorin-e6-TME were prepared by a literature method [36,37,38]. Et_3_N (63.5 µL) was added to a stirred solution of 3-(3-bromopropoxy) chlorin-e6-TME (276 mg, 0.078 mmol) in CH_2_Cl_2_ under a N_2_ atmosphere. To the mixture cooled at 0 °C, the solution of 1-thio-β-d-tetraacetylmannose (3.2 equiv.) in CH_2_Cl_2_ was added dropwise. After being stirred for 3 h at room temperature under a N_2_ atmosphere, the mixture was transferred to a separating funnel, then CH_2_Cl_2_ and water were added, and the organic layer was separated. The aqueous layer was washed with brine and dried over sodium sulfate, then evaporated to dryness. The residue was purified by column chromatography (CH_2_Cl_2_/AcOEt, 1:2) to give acetylated β-M-Ce6. Deprotection was carried out in the ordinary way. NaOMe/MeOH suspension (10 equiv.) was added to acetylated β-M-Ce6 (0.152 mmol) in dried MeOH under the N_2_ atmosphere. After being stirred for 0.5 h at room temperature, the solution was then quenched by AcOH (100 µL) and the mixture was evaporated to dryness. The residue was purified by column chromatography (CH_2_Cl_2_/AcOEt, 10:1) to give β-M-Ce6. ^1^H NMR (400 MHz, CDCl_3_): δ = 9.78 (d, *J* = 13.2 Hz, 1H), 9.67 (d, *J* = 5.2 Hz, 1H), 8.69 (d, *J* = 2.4 Hz, 1H), 5.85–5.86 (m, 1H), 5.20–5.40 (m, 4H), 4.35–4.50 (m, 2H), 4.20 (s, 3H), 3.85–3.65 (m, 6H), 3.54–3.60 (m, 11H), 3.40–3.50 (m, 2H), 3.42 (s, 3H), 3.29 (s, 3H), 2.90–3.20 (m, 2H), 2.30–2.80 (m, 7H), 2.20–2.05 (m, 3H), 1.65–1.80 (m, 8H), −1.55 (s, 2H). MALDI-TOF-MS: (C_46_H_60_N_4_O_12_S) calcd. 892.39; found. 892.37.

### 4.2. Cell Culture

Human glioblastoma U251 cells (Riken Cell Bank, Tsukuba, Japan) were cultured in 100 mm cell culture dishes (Thermo Scientific, Waltham, MA, USA) and 96-well plates (Thermo Scientific, Waltham, MA, USA) in Dulbecco’s modified eagle medium (DMEM, Nissui, Tokyo, Japan) supplemented with 10% fetal bovine serum (Nichirei Bioscience, Tokyo, Japan) at 37 °C with 5% CO_2_, as described previously [39]. Cells were seeded at 1 × 10^4^ cells/well and cultured for 24 h before all experiments, except for the photosensitizer transport assay. Experimental protocols were approved by the Regulations for Biological Research at Tokyo University of Pharmacy and Life Sciences.

### 4.3. PDT

PDT was performed as described previously with small modifications [40]. The stock solutions of each photosensitizer were prepared with saline for TS (Meiji Seika Pharma, Tokyo, Japan) and dimethyl sulfoxide (DMSO) for other photosensitizers. For the dose dependency test, cultured cells were treated with 0–62.5 µM TS, 0–156.3 nM β-G-Ce6, or 0–187.5 nM β-M-Ce6 in fresh medium for 1 h. For the time dependency test, cells were treated with TS (62.5 µM), β-G-Ce6 (78.1 nM), or β-M-Ce6 (93.8 nM) in fresh medium for 5–180 min. Immediately after washing with PBS, cells were subjected to laser irradiation (wave length: 664 nm; laser power: 3.4 mW/cm^2^; total dose of laser irradiation: 1 J/cm^2^) using a semi-conductor laser irradiator, ZH-L5011HJP (Meiji Seika Pharma, Tokyo, Japan).

### 4.4. Cell Viability Assay

The cell viability assay was performed as described previously [41]. Twenty-four hours after laser irradiation, cell viability was measured using Cell Counting Kit-8 (Dojindo, Kumamoto, Japan). The Cell Count Kit-8 solution was mixed with culture medium at a final concentration of 10% and the mixture was applied to PDT-treated cells for 1 h at 37 °C. After incubation, absorbance was measured at 450 nm using a Varioskan Flash microplate reader (Thermo Scientific, Waltham, MA, USA).

### 4.5. Measurement of the Partition Coefficient

Both β-M-Ce6 and β-G-Ce6 were dissolved individually in DMSO to prepare stock solutions (5 × 10^−3^ M). The stock solution was diluted with a 1:1 1-octanol/acetone mixed solution, which was characterized by UV-vis spectroscopy (V-500 spectrophotometer, JASCO, Tokyo, Japan) to determine the molar absorbance coefficient in the 1:1 1-octanol/acetone-mixed solution. Similarly, the molar absorbance coefficient in a 1:1 PBS/acetone-mixed solution was determined. A 1:1 1-octanol/PBS-mixed solution was prepared. From the solution, the 1-octanol phase (600 μL) and PBS phase (600 μL) were collected and combined. A stock solution (3 μL) was added, mixed using a vortex mixer, and then centrifuged to obtain the partition solution. The 1 − *octanol* phase (500 μL) was collected from the partition solution and diluted with 1-octanol (500 μL) and acetone (1.00 mL), which was characterized by UV-vis spectroscopy to determine the concentration in the 1:1 1-octanol/acetone-mixed solution ([*C*_1−*octanol*_]). Similarly, the PBS phase (500 μL) was collected from the partition solution and diluted with PBS (500 μL) and acetone (1.00 mL), which provided the concentration in the 1:1 1-PBS/acetone-mixed solution ([*C_PBS_*]). The *log*([*C*_1−*octanol*_]/[*C_PBS_*]) values were calculated to provide the *log P*-values.

### 4.6. Photosensitizer Transport Assay

For fluorescence microscopy, U251 cells were cultured on 8-chamber glass-bottomed dishes coated with 0.04% Polyethyleneimine (Merck, Darmstadt, Germany) at 3 × 10^4^ cells/well. Cells were cultured for 24 h before the transport assay. Precooled (4 °C) or prewarmed (37 °C) fresh HEPES containing DMEM without phenol red (FUJIFILM Wako Pure Chemical, Osaka, Japan) with 2 µM photosensitizer was applied to the culture for 30 min at 4 or 37 °C. Cells were washed twice with PBS and fixed with 4% paraformaldehyde (Merck, Darmstadt, Germany) in 0.1 M phosphate buffer for 30 min at room temperature. Fluorescence of the photosensitizer was observed under an Eclipse Ti-U inverted microscope (Nikon, Tokyo, Japan) equipped with a filter cube (Ex: 340–380 nm; DM: 400 nm; Em: 672–716 nm) and CMOS Zyla5.5 camera (Andor technology, Belfast, UK). Fluorescence data were collected and processed by NIS-elements (Nikon, Tokyo, Japan) and Photoshop CS6. Fluorescence intensities were measured by ImageJ 1.52k. Data were analyzed by Excel for Mac 2016.

### 4.7. Photosensitizer Distribution Assay

U251 cells were cultured on eight-chamber glass-bottomed dishes coated with 0.04% PEI at 3 × 10^4^ cells/well. Cells were cultured for 24 h before the distribution assay. CytoPainter Golgi staining kit Green (200×, Abcam, Cambridge, UK), MitoBright LT Green (50 nM, Dojindo, Kumamoto, Japan), and Lysotracker Green (200 nM, Thermo Scientific, Waltham, MA, USA) were used in accordance with the manufacturers’ protocols to stain the Golgi apparatus, mitochondria, and lysosomes, respectively. Briefly, fresh medium containing each dye and 10 µM photosensitizer was applied to the cells for 30 min at 37 °C with 5% CO_2_. The cells were washed twice with PBS, and medium was replaced with HEPES containing DMEM without phenol red. Fluorescence of each organelle marker and the photosensitizer were obtained and processed by the same methods described above using a filter cube GFP-B for organelle markers (Ex: 460–500 nm, DM: 505 nm; Em: 510–560 nm) and photosensitizers (Ex: 340–380 nm; DM: 400 nm; Em: 672–716 nm).

### 4.8. Statistical Analysis

If not stated otherwise, data are expressed as the mean ± SEM. Differences between two datasets and multiple datasets were assessed using the Student’s *t*-test and one-way analysis of variance (ANOVA) with the Tukey–Kramer post-hoc test, respectively. All data were collected and analyzed using a double-blinded approach.
